# College Note

**Published:** 1898-10

**Authors:** 


### COLLEGE NOTE.

MEDICAL COLLEGE CONSOLIDATION.—There is a growing tendency, and one that should be encouraged, throughout the country to a union of two or more weakly medical colleges in any one city and the formation from them of one strong institution. Nashville, Buffalo and New York have led in this reform (though the latter has gained nothing in the way of reducing the number of schools, merely preventing an increase), and now there is talk of consolidation in Columbus, O. The *Columbus Medical Journal* says that a proposition had been made looking to a union of the Ohio Medical University and Starling Medical College as the medical department of the Ohio State University. There are some objections, sentimental and financial, urged against the proposed foundation of the medical department of the State University out of the remains of the two existent schools, but the advantages are patent and should outweigh all objections to the scheme.—*Medical Record*, September 24, 1898.

Our esteemed contemporary might have added Atlanta, Ga., to the list.

